# Views of children and young adults about Whole Genome Sequencing in newborn screening: a qualitative study

**DOI:** 10.1038/s41431-024-01614-x

**Published:** 2024-06-19

**Authors:** Molly Parfett, Faye Johnson, Rebecca Bennett, Fiona Ulph

**Affiliations:** 1 Manchester Centre for Health Psychology, Division of Psychology & Mental Health, School of Health Sciences, Faculty of Biology, Medicine and Health, University of Manchester, Manchester Academic Health Science Centre, Oxford Road, Manchester, M13 9PL UK; 2 Centre for Social Ethics and Policy, Department of Law, School of Social Sciences, Faculty of Humanities, University of Manchester, Oxford Road, Manchester, M13 9PL UK

**Keywords:** Health policy, Social sciences

## Abstract

Whole Genome Sequencing (WGS) in newborn screening is ethically complex. Parents will provide proxy consent for initial participation and 16-year-olds will be approached to consent to continued storage of their genome. We explored the views of 11–25-year-olds to understand the information needs of this age group and the views of the next generation of parents. This two-phase UK study involved: a secondary analysis of focus groups with young adults and a diary and focus group study with children with CF. Diaries were analysed using content analysis, focus group data were analysed using reflexive thematic analysis. Diaries illustrated how children formed genomic knowledge and their questions. Participants broadly supported WGS-NBS based on a belief that all results improve health. Pre-study knowledge was sometimes correct, other-times it drew on vicarious ideas that could cause distress. Children showed an ability to appreciate the complexity of deciding which results should be returned. Focus groups: All participants counterbalanced the benefits and risks of WGS-NBS. Children demonstrated innate trust in doctors, whereas young adults wanted parent-doctor collaboration in decision-making. Young adults conditionally supported WGS-NBS depending on treatability, severity, onset and consent. Children wanted parents to be informed of a broader range of results, but valued informed choice too. More research is needed to understand healthy children’s views. Although small samples, this work provides insight into the understanding and concerns of young adults and children which could help when trying to discuss this topic with them.

## Introduction

Whole Genome Sequencing (WGS) is being piloted in NBS (WGS-NBS) to increase diagnosis and treatment of rare conditions [1]. This is ethically challenging [2], raising questions about which results are reported and their impact [3, 4]. Incorporating societal views into WGS-NBS design is crucial to ensuring people find WGS-NBS acceptable and minimising harm [5, 6].

Parents value WGS-NBS’s early diagnosis and treatment, but have concerns about psychological distress, removing children’s autonomy, data storage and privacy, adult-onset and uncertain results [7–10]. There is conditional public support for WGS-NBS [6]. Conditions for accepting this included screening for clinically-actionable, childhood-onset conditions only, providing genetic counselling, and health-professional training [6].

Research with parents shows most plan to tell their children their NBS results in childhood, however, some postpone disclosure due to perceived negative impacts on children’s self-esteem and confidence [11–13] or parents’ low confidence about what and when to tell their children [12, 14]. Exploring children’s views of WGS-NBS should help parents understand how to support their children and will become more pressing when WGS-NBS pilots require 16-year-olds to be contacted regarding the continued storage of their genomes. Nascent research with 13–18-year-olds [15] and 11–19-year-olds with rare diseases [16] suggests they have independently formulated views, were able to express their preferences, and wanted their views involved in care. This study aims to explore children’s and young adults’ views and sense-making of WGS-NBS. This should help parents understand when and how best to scaffold novel WGS-NBS onto existing knowledge and help ensure that the views’ of the next generation of parents are known to policy-makers.

## Methods

A two-phase qualitative study design was used to facilitate in-depth insight into participants’ views. First, healthy young adults’ views (HYAs) towards WGS-NBS were explored, followed by children with CF’s views. The HYA phase explored their pre-existing views on pharmacogenetic testing (not reported here) and WGS-NBS via focus groups (FGs). This enabled us to gain insight into HYA’s concerns and knowledge and use this to design, with the CF Trust, a virtual FG study exploring children’s WGS-NBS views. Virtual FG with CF participants have been used previously [17], although they risk connection issues disrupting discussions [18]. Prior to the FG children were asked to engage with a module and diary reflection task. This enabled participants to submit individual, confidential responses and ask questions [19] about topics and engage with information provided over a longer time period. Through this process and receiving e-mails from the researchers sending the next diary entry they were able to build a relationship with the researchers and learn that any response and question was valid before FG participation.

### Sampling

#### HYAs

Thirty-four 18–25-year-olds were recruited from a psychology course in England and compensated with course credits; standard compensation in psychology courses.

#### Children with CF

Participants aged 11–15 were recruited from the CF Trust’s support groups which ensured the children knew each other and were used to discussing CF-related topics, thus minimising harm. CF Trust staff emailed information sheets for children and parents/guardians. Upon contacting the researcher, parents/guardians were sent the first diary sheet; return implied consent/assent. Parental consent and child assent were gained for the FG. Participants received a £50 voucher. Six children completed diaries. Four of these participated in the FG. All participants in the support groups were invited, so participant numbers were dictated by support-group size and response rate.

### Data collection

#### HYAs

Four FG were conducted in person. The schedule, developed by FU, explored views towards pharmacogenetic testing and WGS-NBS. A slide informing participants about WGS-NBS was shown during the group.

#### Children with CF

Staff from the CF Trust provided guidance on study materials, recruitment and processes.

##### Diary

This was completed over 7 days within two weeks via Qualtrics. Each day participants were asked for their initial thoughts about a topic before being given information the next day. Topics were NBS, WGS-NBS, uncertain results, adult-onset results, and results usage. Participants could raise questions throughout.

##### FG

The schedule was developed post-analysis of HYA data by FU, RB, and FJ. Participants were asked to develop a group definition of WGS-NBS. Any misunderstandings were explored and clarified. Next, participants discussed what results they thought should be reported and why. Finally, participants were shown a timeline representing a lifespan and asked when people should make decisions about their data. The FG lasted 98 min. One participant experienced audio issues; written responses via the chat were verbalised by a researcher.

### Analysis

Diary data were analysed using manifest, inductive, content analysis [20] exploring surface-level responses, but also how children made sense of topics [21]. Responses were split into meaning units and coded. Codes were checked against the entire dataset to ensure a holistic representation Codes were reviewed for dominant patterns and grouped into understanding, positive perceptions and concerns.

Reflexive thematic analysis (RTA) was used to identify and interpret patterns across FGs [22]. An essentialist orientation aimed to report participants’ perspectives, assuming language represented their objective reality [22]. Coding was inductive at the manifest level.

Analysis occurred separately with themes being developed for both the HYA’s and children’s datasets. Following analysis, the visual maps of themes were reviewed and merged. The HYA structure dominates due to children’s shorter responses.

Reflexivity was practiced throughout. The researchers’ beliefs were WGS-NBS provides population-level health benefits, but should be limited to clinically-actionable, childhood-onset conditions, and implemented when sufficient family support is available.

## Results

Thirty-four 18–25-year-olds participated (31 females, 3 males). The children with CF’s characteristics can be seen in Table 1.

**Table 1:** Children with CF’s characteristics.

Pseudonym	Age	Self-rated level of genetic understanding
*Max*	13	Little
*James*	12	Little
*Poppy*	11	Average
*Ben*	15	Little
*Katie*	14	Little
*Alex*	12	Nothing

### Diaries

Responses about WGS given before any information was provided ranged from “a full part of something” (Poppy, youngest child) to “sequencing the genetic code” (Ben, oldest child). One response “changing your genome one step at a time” (James, 12) could cause distress if not amended.

About half the questions children raised before receiving information were seeking to understand what terms meant. About a third were WGS-NBS practicalities i.e. “how does it work?” (James) with one questioning “does it work?” (Katie). After receiving information, there were two questions. One indicated that the child was struggling to understand and would benefit from contact from the research team, the other was:

“How DNA and genetics can be put together into words or a ‘code’, that’s the bit I can’t wrap my head around.” (Katie, 14).

She is one of the older participants. Questions were raised when the term “adult-onset conditions” was introduced, but after receiving information there were no questions. When considering research using genomes, questions were “is the research cost effective?” (Ben) and “is it ok if the child decides?” (James).

#### Positive perceptions

The most common responses either showed general support “I think it’s a good idea” (Poppy) who also indicated diagnoses were valued “good it will show up more things” (Poppy). That WGS-NBS would create benefits via subsequent treatment was also common:

“I think it’s a good idea to know before rather than later because it can help you become well” (Max)

Individual choice was seen as important when considering uncertain, carrier and adult-onset results and genome storage “if there is consent, it would be a good thing” (Katie). The belief that WGS-NBS could be beneficial to research featured in responses before the topic was introduced. Information about carrier results triggered some to value information for reproductive planning, whilst the adult-onset topic caused some to respond that they would value the opportunity to prepare.

#### Concerns

Children were most concerned about results triggering distress i.e. having uncertain results before symptoms “it makes [parents] nervous and worry on a daily basis” (Max) or adult-onset conditions “it could cause unnecessary stress” (Katie). Children appreciated uncertain results are challenging for all “It must be hard for the doctor” (Alex). When considering the return of “non-severe” results, they wanted parents to have a choice about receiving these. Children also weighed up risks of different result types “I think [a carrier result] is better than uncertain results, but it is a risk and if they choose that risk then their child could suffer” (Max).

### FGs

**Fig. 1:**
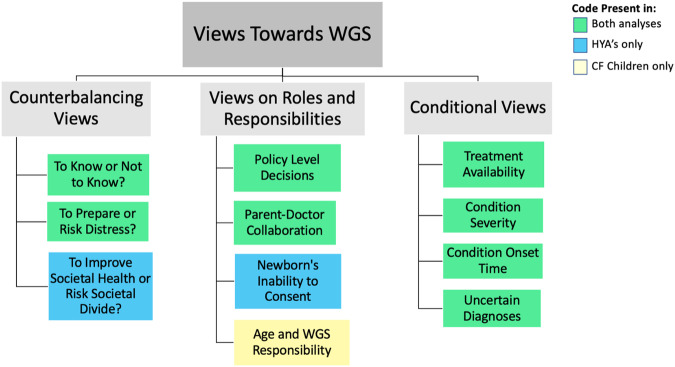
Thematic map illustrating coverage of datasets. Green boxes show codes that were present across both datasets. Blue boxes show codes that were only present in the HYA data. Yellow show codes only present in the CF Children data.

Three themes were created. ‘Counterbalancing Views’: counterbalancing benefits and risks. ‘Roles and Responsibilities’: views, concerns, and preferences towards parents’, doctors’, and policy-makers’ role. ‘Conditional Views’: support for WGS-NBS dependent on certain criteria Figure one demonstrates the overlap and divergence between samples across themes. If codes spanned both datasets “participants” is used, otherwise we specify which dataset codes represent (Fig. 1).

### Counterbalancing views: to know or not? – immediate impact

Most participants reported multiple benefits of knowledge gained via WGS-NBS. Some valued the reassurance this would give parents focusing on the comprehensiveness of genomic information. Participants perceived benefits including treatment opportunities, symptoms explanations and the ability to make informed decisions. These were expressed differently dependent on age:

*If you have a long-term illness that needs treating […] The parent might ask things like why is my child not gaining weight blah, blah. But that could be explained to them and could have like more information and kind of understand it. (Amanda, 21, Female, group A)*

*you can catch it early if it’s something bad that can affect your life so it’s better to like, catch it early. (Max, 13)*

Although Max does not explicitly use the term “treatment”, his phrase “catch it early” is usually used when knowing about something means one can make an alteration to improve an outcome. Two children, of different genders and ages (11 and 13), counterbalanced these views with parents feeling anxious:

*…they would be like ‘oh my god my child’s got something wrong with them’ or something like ‘oh my ga, I’m stressed out’… (Poppy, 11)*

HYAs also counterbalanced views with concerns that, uncertain, untreatable, or mild results had limited use and could cause harm. One worried results may change parenting behaviour, even with a mild diagnosis:

*…if you know something and it might not be that serious you could treat the baby differently maybe not explicitly but in an implicit way. (Georgie, 20, female, group C)*

Further risks described include stigmatisation “…people might see them differently or might call them names…” (Amelia, 18, female, group C) and parents’ distress and burden.

### Counterbalancing views: prepare or risk distress about future?

Additional to how information could affect understanding of *current* symptoms, participants balanced the value of WGS-NBS results enabling people to know something about their future. A benefit was enabling people to prepare, practically and psychologically, for condition-onset “Tell parents everything cos they get the support.” (James, 12). Practical preparations cited by HYAs included lifestyle and environmental changes to delay or prevent onset:

*…maybe if you have the cancer gene like the BRCA 1 or 2 you would just change your lifestyle to accustom to that since you know you might get cancer. (Emily, 20, female, group B)*

When considering how they would feel about WGS-NBS as parents, distress was frequently raised as counter-arguments to pre-symptomatic knowledge. Some did this across the discussion, whilst others did this within one response:

*…if you know that it’s going to happen before it does it might become like a really negative like thing of dread […] but I don’t know, you could argue that if you’ve got time to prepare you’d be able to deal with it more positively…(Aysha, 19, female, group A)*.

Three children acknowledged the potential for distress from unanticipated results:

*…the child, or the family, could take it quite erm, of a panic, and people react differently and some people could be very nervous and start to not do things to, that they do usually…(Max, 13)*

Whilst acknowledging the parental-distress risk, the eldest child suggested that, for children, the benefits of preparation may outweigh this risk. Within this they also valued physical-health outcomes above psychological impact:

*It might upset the parents but at the end of the day it’s more about the child than the parents. (James,12)*

One HYA appreciated that making changes to optimise health were not always in parents’ control and this could cause concern:

*…there might be an underlying genetic basis for the illness, but perhaps its more the environment, the parent might become really stressed that if they do this that’s going to bring it [the condition] up…(Afia, 19, female, group C)*

Whilst the opportunity to prepare for condition onset was valued by many HYAs and all children this illustrates how most participants appreciated this was not beneficial for all.

### Counterbalancing views: increase societal health or divide?

Societal implications of WGS-NBS were raised unprompted by HYAs. Some viewed WGS-NBS as an opportunity to improve population health:

*I actually think it would be worth it getting everyone tested because it seems to suggest that it can prevent preventable diseases (Gemma, 18, female, group C)*

Others counter-argued, expressing a fear of exacerbating health inequalities if those with access to private healthcare can act on WGS-NBS results, but others cannot.

*“…it may only be the people who have the ability to pay for private healthcare or have more access and money to treatments and then, the poorer families who know their children are going to end up with this, there’s nothing they can do about it. So, there might be some kind of massive divide. (Elena, 21, female, group C)”*

Others described societal divide based on WGS-NBS uptake:

*One group would ask people why they didn’t examine their kids and why didn’t they want to know everything and then the other group will be like “we don’t need to know everything because it’ll make it harder for the families”…(David, 23, male, group B)*

Although some viewed WGS-NBS as beneficial for population health, negative societal implications – including screening-out diversity and increased division – arose more frequently. Children and one HYA group did not discuss this topic.

### Roles and responsibilities: policy-level decisions

When considering which WGS-NBS results to report, HYAs thought individual healthcare professionals should not choose. Inherent in responses was an appreciation of the power imbalance this created in the therapeutic relationship:

*…if you’re keeping some [genetic information] and the doctors know something, they’re always playing God in terms of like what the parents know (Elena, 21, female, group C)*

Whether this situation would arise in reality, it highlights a concern held by participants and could explain why HYAs wanted policy-level decision enacted across healthcare to avoid subjectivity and ensure fairness. Importantly they linked this to screening acceptability:

*…it’s an issue of fairness, who gets to make the decision like I kinda agree it should be standardised one way or another otherwise there’ll be so much backlash…(Aysha, 19, female, group B)*

Children preferred doctors to decide. Implicit in their responses was trust that doctors know best what to reveal to patients and that improved medical-outc*omes will following result disclosure*.

*the doctors should know better, so like if they think it’s better that they don’t bring it up then they shouldn’t…(James, 12)*

*leave the doctors to decide whether it’s serious enough to tell the patient. And if it is, then at least they know and they can do stuff to improve, and how to get rid of it. (Max, 13)*

Children described a doctor’s responsibility to inform parents of notable results to avoid parental shock, retain trust, and facilitate preparation. Thus, both age groups spontaneously considered the negative consequences of the public believing results were not disclosed appropriately, albeit they came up with different ways of solving this.

#### Roles and responsibilities: parent-doctor collaboration

Although HYAs believed policymakers should determine the results returned to parents, they wanted doctor-parent collaboration regarding whether the baby has WGS-NBS. Most felt the parent should make the ultimate decision with doctors providing advice regarding possible outcomes:

*“[the parents] are going to have to raise it [the child] and answer to the child later, the health professionals should definitely advise but ultimately it should be up to the parents.” (Sally, 19, female, group D)*

Participants in one group believed doctors should be able to overrule parents in the child’s best interests—citing refusing medical intervention due to religious beliefs or neglect. When one participant was asked what should happen if parents decide against WGS-NBS for religious reasons, their response shows how this medical intervention is warranted in clear cases of harm, which may not apply to all results returned by WGS-NBS:

*I think the doctors can take the lead in that case. […] there are some things that 100% work and are really positive but then the only thing holding the baby back and could kill them is whether the parents will consent to it. (Priya, 20, female, group A)*

When children discussed parents’ and doctors’ roles they viewed the roles as distinct. Doctors diagnose and provide parents with information regarding their child’s genetic vulnerability; parents comprehend information and act accordingly.

### Roles and responsibilities: newborn’s inability to consent

All HYA groups raised the rights of the individual having WGS-NBS (the newborn) unprompted. Some reflected on the issue that parents would be making decisions, which impact them as adults:

*…if it was me if I, maybe I don’t want to know about certain stuff but I wouldn’t get a choice because my parents would have tested me when I was a baby. (Hannah, 22, female, group B)*

The individual’s inability to consent was seen as unproblematic by some. The notion that parents’ beliefs would likely be adopted by their child was raised by participants in two separate groups. Again this focused on clear-cut cases, rather than the nuances presented in WGS-NBS:

*…if their parent had this religious belief then the child is quite likely- I mean obviously people can stray from their religion, but quite likely to hold these belief systems themselves…(Holly, 19, female, group A)*

This tension between the individual and their parents was not raised in the children’s group.

#### Roles and responsibilities: age and WGS responsibility

Due to the above, the researchers wanted to explore children’s opinions about what age they take responsibility for their WGS-NBS information. Children’s views varied. Initially, two felt their current age [11–13] was too young and the three youngest participants thought 16–18 years old was ideal:

*…our age now is a bit too young cos we’re not fully matured, erm, I think erm it should be at least 16-18 when you’re more, more sensible…(Max, 13)*

The eldest child participant, who often offered counter-arguments, felt that 30-years-old was more appropriate. The belief that doctors know best was, again, expressed here:

*I think people should only really be told when they’re about 30, or given control over their medical things when they’re about 30. The doctors should handle it before. (Ben, 15)*

They explained that 20-year-olds do not prioritise health and intimated there was a burden inherent in deciding about your genome:

*…most people in their 20* *s tend to go to parties or university, they either don’t want to or don’t have time to deal with important things*

Children recognised that maturity and intelligence, factors deemed important when taking responsibility for their genome, did not correspond with age:

*I think cos we’re all different, we all do different things and we don’t all understand things, but some of us do. So it depends on if they’re willing to, and if they can. (Max, 13)*

Throughout the discussion with children, the role of individual differences arose frequently regarding multiple aspects of WGS-NBS.

### Conditional views: treatment-availability

HYAs had a caveat that WGS-NBS results should lead to treatment and wanted to avoid knowing results without clear treatments. Some reflected from a personal stance whilst others did so from the family perspective:

*…think about what those families might think if they knew they had a child that was going to develop something and there was absolutely nothing they could do…(Elena, 21, female, group C)*

All children agreed that both treatable and untreatable results should be available: “I agree [all results should be returned], so that you can know what is expected.” (James,12).

Clear disparities existed between HYAs and children’s views.

### Conditional views: condition-severity

HYAs also had the caveat that WGS-NBS reported only severe conditions with some being clear what constitutes severe:

*if it’s life threatening and the doctors know about it then they should definitely inform the parents […] it should depend on the severity of it. (Natasha, 19, female, group D)*

HYAs feared that the return of mild results would cause unnecessary distress. Children wanted more results provided to parents. One reflected on a pet allergy arguing it could be useful to know about before getting a pet. The only exception to this was if the condition would not affect the child whatsoever. One child defined this as a condition that would not cause pain, nor make the child feel abnormal, or limit daily activities.

*…if it’s not gonna affect them in the slightest then I don’t think they really need to know. But if it’s gonna affect them slightly then it’s always good to know in case it builds up. (Max,13)*

Whether these views are being driven by their age, or their illness experience of regular hospital appointments was not clear.

### Conditional views: condition-onset

HYAs did not want results for late adulthood conditions, discussing Alzheimer’s and dementia unprompted.

*“…if I found out I had like, I don’t know a predisposition for Alzheimer’s or something I would really not want to know right now at all.” (Hannah, 22, female, group B)*

Regarding late-onset conditions, all HYA groups felt that unnecessary worry outweighed perceived benefits. Conversely, onset did not influence children’s views. Three children believed results for both adult- and child-onset conditions should be returned to parents to avoid concealing results. James preferred results were returned if symptoms emerged to avoid parents overreacting. He did, however, also value the parents’ rights: “if the parents wanna know then you should maybe consider telling them…” (James, 12) suggesting a case-by-case approach which was also supported by the next eldest child.

### Conditional views: certainty

If a newborn would definitely develop the condition, HYAs wanted results available. For an uncertain diagnosis, all HYAs voiced concerns about triggering undue worry:

*“if there’s only a small chance of them getting it I would rather wait until it actually happened rather than spending ages worrying about it.” (Ella, 20, Female, group D)*

Similarly, HYAs did not want genetic predisposition results. One participant specified common conditions influenced by lifestyle, citing cancer and dementia, as it provided limited additional information. The negative psychological impact of receiving uncertain results, or those regarding genetic predispositions, outweighed the benefits for many HYAs. Some children believed all results should be returned to avoid concealment and distress:

*I think they should be told because then they can prepare for the worst. Cos if they’re not told, and it does happen, it’s a sudden shock. (Max, 13)*

Children’s views towards previously discussed results remained constant throughout their discussion. However, during discussion of uncertain results, two children changed their views and wanted parental choose regarding receiving uncertain results “I think it’s okay if you want to know, but if you don’t want to see then it’s okay.” (James, 12).

## Discussion

We found that participants held complex views about WGS-NBS, seeing neither exclusively positively or negatively. Their views evolved during discussions. HYAs demonstrated greater personal conflict and complexity within their views as discussions evolved and considered societal benefits and risks of WGS-NBS. Whilst children also counterbalanced perceived benefits and risks, they were more optimistic [15, 16] and their views more stable.

Participants valued early diagnosis and treatment, psychological and practical preparation, and improving prognosis. Risks included unnecessary parental distress and treating children differently. These views align with those expressed by parents [7–9], the public [6], and children with rare diseases [16]. Participants focused more on how parents may view their child differently, than results altering how the screened individual views themselves [15]. The timing of NBS results may explain this. Data storage concerns found previously [7–9] were only raised by one HYA in line with research with this age group [16].

HYAs thought results should be limited to certain, treatable, childhood-onset, severe conditions, in line with the public’s [6] and parents’ views [7–9]. Children were more concerned about concealment and later shock and wanted a broader range of results disclosed. Our work may explain this as we inferred from responses that children believed that not reporting all information is deception, rather than potentially avoiding harm.

HYAs wanted policy to standardise results returned and avoid subjectivity. This extends previous findings where participants wanted policy-level involvement in WGS-NBS to avoid data privatisation [6]. Children’s views on this topic differed to HYAs. Children’s trust in doctors was mentioned repeatedly [23]. Pandemic portrayal of doctors as superheroes [24] and/or their own dependence on doctors might explain this. Alternatively, the concept of healthcare policy may not yet be salient for children who instead use the word “doctors” to represent all healthcare actors. Children highlighted the importance of considering individual differences (e.g. age and maturity) when making decisions as found in an older age group previously [15].

We found HYAs preferred parent-doctor collaboration in NBS decision-making, similar to a previous study with 13–18 year olds which found they wanted collaboration between parents–child and similarly found that participants highlighted the removal of their right to an open future as problematic [15]. Children in the current thought ~16 years (the proposed age of re-consent in UK) was suitable to make decisions about your genome, whilst others felt this should be much older.

This study’s strength is the involvement of a multi-disciplinary, multi-agency team in the design creation enabling novel insights. Following previous research [6], children were given age-appropriate information to consider, avoiding challenges seen previously where limited knowledge curtailed participants’ ability to express opinions [7]. Our design enabled views to be gathered sensitively and monitoring to ensure misunderstandings did not cause distress. One child experiencing confusion was able to raise this, and we scaffolded an explanation for them. HYAs participated first to assess feasibility and enabling more sensitivity when working with children. Audio failure meant one child gave text responses. As a researcher verbalised their responses and checked meaning with them, these were included in the analysis. Caution in interpreting these results is warranted as participants were responding to hypothetical scenarios, which has been shown to elicit more positive views [15]. Furthermore, most of the HYA were females, suggesting a need for further research with males.

Children demonstrated greater variations in understanding and articulation resulting in some voices being presented more frequently than others. However, enabling this age group to participate is crucial as they are the age when parents may start talking to their children about NBS results [12] and are approaching the age when WGS-NBS screened individuals will be contacted about continued storage of their genome. Whilst this study provides some insight into children with CF’s views, further research is urgently needed to understand how best to talk to, and support, children and young adults to make decisions about their genome’s storage and access. This study shows that children can engage in such debates.

## Data Availability

The datasets generated and/or analysed during the current study are available from the corresponding author on reasonable request.
